# Five-Feature Model for Developing the Classifier for Synergistic vs. Antagonistic Drug Combinations Built by XGBoost

**DOI:** 10.3389/fgene.2019.00600

**Published:** 2019-07-09

**Authors:** Xiangjun Ji, Weida Tong, Zhichao Liu, Tieliu Shi

**Affiliations:** ^1^The Center for Bioinformatics and Computational Biology, Shanghai Key Laboratory of Regulatory Biology, Institute of Biomedical Sciences–School of Life Sciences, East China Normal University, Shanghai, China; ^2^Guangdong Provincial Key Laboratory of Proteomics, School of Basic Medical Sciences, Southern Medical University, Guangzhou, China; ^3^National Center for Toxicological Research, United States Food and Drug Administration, Jefferson, AR, United States; ^4^National Center for International Research of Biological Targeting Diagnosis and Therapy/Guangxi Key Laboratory of Biological Targeting Diagnosis and Therapy Research/Collaborative Innovation Center for Targeting Tumor Diagnosis and Therapy, Guangxi Medical University, Nanning, China

**Keywords:** drug combination, XGBoost classifier, synergistic drug pair, antagonistic drug pair, model performance

## Abstract

Combinatorial drug therapy can improve the therapeutic effect and reduce the corresponding adverse events. *In silico* strategies to classify synergistic vs. antagonistic drug pairs is more efficient than experimental strategies. However, most of the developed methods have been applied only to cancer therapies. In this study, we introduce a novel method, XGBoost, based on five features of drugs and biomolecular networks of their targets, to classify synergistic vs. antagonistic drug combinations from different drug categories. We found that XGBoost outperformed other classifiers in both stratified fivefold cross-validation (CV) and independent validation. For example, XGBoost achieved higher predictive accuracy than other models (0.86, 0.78, 0.78, and 0.83 for XGBoost, logistic regression, naïve Bayesian, and random forest, respectively) for an independent validation set. We also found that the five-feature XGBoost model is much more effective at predicting combinatorial therapies that have synergistic effects than those with antagonistic effects. The five-feature XGBoost model was also validated on TCGA data with accuracy of 0.79 among the 61 tested drug pairs, which is comparable to that of DeepSynergy. Among the 14 main anatomical/pharmacological groups classified according to WHO Anatomic Therapeutic Class, for drugs belonging to five groups, their prediction accuracy was significantly increased (odds ratio < 1) or reduced (odds ratio > 1) (Fisher’s exact test, *p* < 0.05). This study concludes that our five-feature XGBoost model has significant benefits for classifying synergistic vs. antagonistic drug combinations.

## Introduction

The *de novo* drug discovery paradigm of “one drug, one target, and one disease” has been greatly challenged by the increasing rate of drug attrition in clinical trials and drug withdrawal due to severe adverse drug reactions (ADRs) at the post-marketing stage (Wood, 2006). Considering the complexity of disease etiology and pathogenesis, alternative drug development approaches such as drug combinations have been promoted to provide more effective and safer regimens (Flemming, 2014; Sarah, 2017). Combinatorial drug treatments could work synergistically to boost efficacy, or act additively or antagonistically to alleviate ADRs (Jia et al., 2009). Drug combinations have been widely used to counter drug resistance in cancer therapy (Webster, 2016). One example of this is the combination of docetaxel with two HER2 inhibitors (i.e., pertuzumab and trastuzumab) for treating HER2-positive metastatic breast cancer, which achieved an approximately 16-month improvement in overall survival (OS) compared with the conventional single treatment option (Swain et al., 2015). Synthetic lethality could be employed when discussing feasible therapeutic strategies for treating gastric cancer (Guo et al., 2017). Besides oncological drug development, the use of drug combinations is also a popular approach for antibacterial and antifungal therapy (Spitzer et al., 2011) and diabetes (Lu et al., 2017; Xu et al., 2017). For example, Hsp90 inhibitors and the antifungal drugs azoles were combined to treat patients infected with *Candida albicans* and *Saccharomyces cerevisiae* (Hill et al., 2013). As mentioned above, the use of drug combinations has also been applied to alleviate ADRs. One example is fixed-dose combination therapies for treating type 2 diabetes, which effectively eliminated the side effects of diabetes drugs such as cardiovascular toxicity and enhanced the efficacy (Bell, 2013).

Recent success in drug combinations has primarily been the result of serendipity or clinical observation, which is time-consuming and knowledge-driven (Foucquier and Guedj, 2015). Computational approaches offer a rational and exhaustive exploration of all possible drug combination opportunities by integrating different biomedical data profiles (Sun et al., 2013; Bulusu et al., 2016). Efforts have been made to develop *in silico* approaches to accelerate effective drug combination discovery. These computational approaches are mainly divided into three categories: transcriptomic profiles and cell-based drug sensitivity assay-based modeling, network/system biology-based approaches, and machine learning algorithms. For example, Preuer et al. (2018) developed a deep learning modeling named DeepSynergy to predict anti-cancer drug synergy by incorporating chemical and genomic data, yielding an AUC of 0.90. In addition, the predictive performance of DeepSynergy was also superior to that of other state-of-the-art methodologies, including random forest (RF), gradient boosting machine, support vector machine, and elastic net. The pros and cons of these *in silico* approaches have been intensively discussed elsewhere (Bulusu et al., 2016).

Questions have been raised about how to integrate the diversity of biological information into a framework to improve the performance of tools for predicting the efficacy of drug combinations. First, the current *in silico* drug combination models are mainly focused on the field of oncology (Sun et al., 2015; Preuer et al., 2018). There is thus a lack of *in silico* models to explore the opportunities for using drug combinations in other therapeutic categories such as pediatric and infectious diseases. Second, numerous accumulative biological datasets have been generated and become widely available, so a comprehensive assessment of the predictive power of diverse biological profiles is imperative to provide useful information for further model development. Finally, no approach at *in silico* modeling will provide universally valid results. Therefore, we need to carefully define the domain in which modeling results are applicable to maximize their utility. To address these unresolved issues, there is an urgent need for novel methodologies and model development strategies.

XGBoost as a machine learning algorithm has become well established in the machine learning community and gained a positive reputation through numerous machine learning challenges (Chen and Guestrin, 2016). XGBoost is an ensemble method based on gradient boosted trees. Considering the rationale behind XGBoost, it may be a promising algorithm to integrate diverse biological information seamlessly and yield satisfactory predictive results. To the best of our knowledge, the XGBoost methodology has not been applied to classify synergistic vs. antagonistic drug combinations.

In this research, the XGBoost methodology is intended to classify synergistic vs. antagonistic drug combinations. To investigate the potential for applying the XGBoost methodology, we employed five different data profiles, namely, chemical structure information, human phenotypic information, pathways, protein targets, and protein–protein interactions, for model development. The proposed XGBoost model was comprehensively assessed based on feature importance, performance metrics, and degree of overfitting. The model was also compared with state-of-the-art machine/deep learning algorithms including RF, logistic regression (LR), naïve Bayes (NB) classifier, and DeepSynergy. The domains to which the proposed XGBoost model is applicable were also investigated by ranking model performance across different therapeutic categories.

## Materials and Methods

**FIGURE 1:**
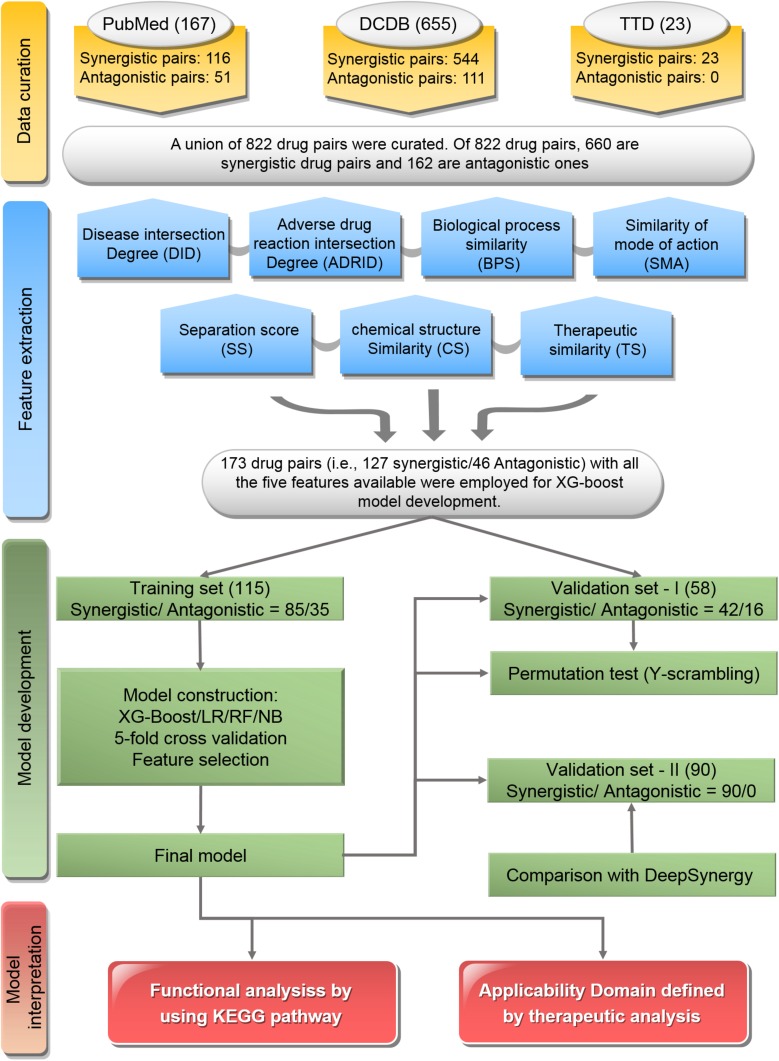
Flowchart of the study: The workflow includes data curation, feature extraction, model development, and model interpretation.

The workflow of this study was illustrated in Figure 1, which included major four parts: data curation, feature extraction, model development, and model interpretation.

### Data Curation

To curate the drug pairs with known combination effectiveness, three data resources including the Drug Combination Database (DCDB) (Liu et al., 2014), Therapeutic Target Database (TTD) (Zhu et al., 2010), and the literature in PubMed (Fiorini et al., 2017) were used.

The DCDB^1^ is devoted to the research and development of multi-component drugs (Liu et al., 2014). The updated DCDB 2.0 collected 1,363 drug combinations (330 approved and 1,033 investigational, including 237 unsuccessful usages), involving 904 individual drugs and 805 targets. In this study, the combinatorial medical effectiveness of 655 drug combinations corresponding to 544 synergistic drug pairs and 111 antagonistic ones was retrieved from DCDB.

Therapeutic Target Database^2^ is a database to provide information about the known and explored therapeutic protein and nucleic acid targets, the targeted disease, pathway information, and the corresponding drugs directed at each of these targets. It contains 75 drug combinations. In this study, the combinatorial medical effectiveness of 23 drug combinations (e.g., 23 synergistic drug pairs vs. 0 antagonistic ones) were employed.

PubMed^3^ comprises more than 28 million citations for the biomedical literature from MEDLINE, life science journals, and online books (Suarez-Almazor et al., 2000; Boddy, 2009). In this study, the combinatorial medical effectiveness of 167 drug combinations (e.g., 116 synergistic drug pairs vs. 51 antagonistic ones) was mined from PubMed with the Java library OpenNLP^4^ for text mining (Supplementary Table S1).

Together, a union list of 822 drug pairs with known combinatorial medical effectiveness based on the three resources was obtained. Among them, 660 are synergistic drug pairs and 162 are antagonistic ones (Supplementary Table S2).

### Feature Extraction

A list of seven features to describe the synergistic effect of drug pairs were generated in this study. These seven features were designed to comprehensively cover the molecular and phenotypic characteristics of drugs as well as their on/off targets. The details of these seven features are listed below:

(1) Disease intersection degree (DID): Drug–disease relationships were obtained from DrugBank (Wishart et al., 2018) and TTD (Li et al., 2018). DID represents the proportion of the same indications of two drugs. The higher the DID, the greater the proportion of the same indications of two drugs. The formula of DID is as follows:

(1) $$D\,I\,D_{a,b}=\frac{D_{a}\cap D_{b}}{D_{a}\cup D_{b}}$$

Among these values, *D_a_* and *D_b_* represent the diseases treated by drugs *a* and *b*, respectively.

(2) Adverse drug reaction intersection degree (ADRID): ADRs were obtained from SIDER (Kuhn et al., 2016) and ADReCS (Cai et al., 2015). We defined ADRID as the Jaccard similarity between ADRs between two drugs. ADRID represents the proportion of the same ADRs of two drugs. The formula of ADRID is as follows:

(2) $$A\,D\,R\,I\,D_{a,b}=\frac{A\,D\,R_{a}\cap A\,D\,R_{b}}{A\,D\,R_{a}\cup A\,D\,R_{b}}$$

Among them, *ADR_a_* and *ADR_b_* represent the ADRs of drugs *a* and *b*, respectively.

(3) Biological process similarity (BPS): BPS indicates the similarity between the biological processes for the interactants of two drugs. The higher the BPS, the greater the similarity of the biological process derived from the targets of two drugs. This feature was measured by GOSemSim (Yu et al., 2010). Targets, enzymes, and transporters of drugs were obtained from DrugBank (Wishart et al., 2018) and DGIDB (Cotto et al., 2018). BPS was calculated in R with the GOSemSim package which can be downloaded from http://www.bioconductor.org/packages/release/bioc/html/GOSemSim.html.

(4) Similarity of mode of action (SMA): This feature indicates the similarity of the mode (promotive/inhibitory) by which drugs act on the target in a drug pair. The higher the SMA, the greater the similarity of the mode (promotive/inhibitory) of action on the target of the two drugs. Drug–target interactions were obtained from DrugBank (Wishart et al., 2018) and DGIdb (Griffith et al., 2013). A protein interactive network with direction was obtained from KEGG (Kanehisa et al., 2016) and SIGNOR (Perfetto et al., 2016). All the interactions were directional and classified as promotive/inhibitory. The mode through which a chemical x acts on another non-adjacent chemical z depends on the relations of chemicals in all the shortest paths from x to z. If there are three chemicals, x, y, and z, with no direct link from x to z:

(a) If x promotes y and y promotes z, then x promotes z;

(b) If x promotes y and y inhibits z, then x inhibits z;

(c) If x inhibits y and y inhibits z, then x promotes z.

Then, the formula of SMA is as follows:

(3) $$A\,M\,S_{a,b}=\frac{\sum_{i=1}^{M}\frac{\sum_{x=1}^{X}c\,{\left(a_{i},b\right)}_{x}}{X}+\sum_{j=1}^{N}\frac{\sum_{y=1}^{Y}c\,{\left(a,b_{j}\right)}_{y}}{Y}}{\sum_{i=1}^{M}\frac{\left|\sum_{x=1}^{X}c\,{\left(a_{i},b\right)}_{x}\right|}{X}+\sum_{j=1}^{N}\frac{\left|\sum_{y=1}^{Y}c\,{\left(a,b_{j}\right)}_{y}\right|}{Y}}$$

*a_i_* and *b_j_* are the targets of drugs *a* and *b*, respectively. *c(a_i_, b)_x_* is the coefficient of the shortest path x from *a_i_* to *b*. The interpretation of *c(a_i_, b)* also applies to *c(b_j_, a)*. If *c(a_i_, b)_x_* = 1, it means that the mode (promotive/inhibitory) of action of drug *b* on the target *a_i_* through path *x* is the same as the mode (promotive/inhibitory) through which drug *a* acts on target *a_i_*. If *c(a_i_, b)_x_* = −1, this means that the mode by which drug b acts on the target *a_i_* through path *x* is the opposite of the mode by which drug *a* acts on target *a_i_*. The numerator is normalized by the denominator in the formula. *SMA_a,b_* ranges from −1 to 1. If the modes by which drug *b* acts on all the targets of drug *a* are the same as the modes by which drug *a* acts on them, *SMA_a,b_* = 1; alternatively, if the modes by which drug *b* acts on all the targets of drug *a* are the opposite of the modes by which drug *a* acts on them, *SMA_a,b_* = −1.

(5) Separation score (SS): This score is initially used to calculate module distances of two diseases, which is referred to as network separation (Menche et al., 2015). We first mapped all drug targets to the protein interaction network from InWeb_IM (Uhlik et al., 2016). In our model, separation score quantifies the network-based separation *S_ab_* of two drugs a and b by comparing the mean shortest distances <*d_aa_*> and <*d_bb_*> between the respective drugs, to the mean shortest distance <*d_ab_*> between their targets:

(4) $$s_{\mathrm{ab}}=<d_{\mathrm{ab}}>-\frac{<d_{\mathrm{aa}}>+<d_{\mathrm{bb}}>}{2}$$

(6) Chemical structure similarity: The simplified molecular-input line-entry system (SMILES) is a specification in form of a line notation for describing the structure of chemical species using short ASCII strings (Weininger, 1988). SMILES information was obtained from DrugBank. Chemical structure similarity was calculated by Tanimoto similarity of SMILES in RDKit (Saubern et al., 2011).

(7) ATC similarity: We used the World Health Organization (WHO) ATC classification system (Skrbo et al., 2004). The ATC similarity between two drugs was induced from Gottlieb et al. (2012).

The calculated features were listed in Supplementary Table S2.

### Model Development

#### The XGBoost Classifier

XGBoost (Extreme Gradient Boosting) is a machine learning technique for regression and classification problems based on the Gradient Boosting Decision Tree (GBDT) (Chen and Guestrin, 2016). The XGBoost model has been widely applied in all kinds of data mining fields for regression and classification, but has not yet been imported into the field of pharmacology. XGBoost is essentially an ensemble method based on gradient boosted tree (Friedman, 2001). In the regression tree, the inside nodes represent values for an attribute test and the leaf nodes with scores represent a decision. The result of the prediction is the sum of the scores predicted by K trees, as shown in the formula below:

(5) $${\hat{y}}_{i}=\sum_{k=1}^{K}f_{k}\,\left(x_{i}\right),f_{k}\in F$$

where *x_i_* is the *i*-th training sample, *f_k_(x_i_)* is the score for the *k*-th tree, and F is the space of functions containing all regression trees. The objective function to be optimized is given by the following formula:

(6) $$\mathrm{obj}\,\left(\theta \right)=\sum_{i=1}^{n}l\,\left(y_{i},{\hat{y}}_{i}\right)+\sum_{k=1}^{K}\Omega \,\left(f_{k}\right)$$

The former $\sum_{i=1}^{n}l\,\left(y_{i},{\hat{y}}_{i}\right)$ is a differentiable loss function that measures whether the model is suitable for training set data. The latter $\sum_{k=1}^{K}\Omega \,\left(f_{k}\right)$ is an item that punishes the complexity of the model. When the complexity of the model increases, the corresponding score is deducted.

In this study, variables input into the XGBoost classifier are the features of drug pairs and the variables that are output are the predicted classes and the corresponding possibilities of combinatorial medical effectiveness in a scale of 0∼1. The probability over 0.5 indicates that the combination is inclined to be synergistic, and the one under 0.5 means that the combination is inclined to be antagonistic. Some prediction values of drug combinations are around 0.5, which reflect that the combination is inclined to be additive.

#### Model Generation

(1) Division of training set and independent validation set: Of the 822 drug pairs curated with known combinatorial medical effectiveness, 173 drug pairs (synergistic drug pairs: antagonistic drug pairs ratio = 127:46) contain all the seven features described above were used for model construction and comparison since other models built by other classifiers (LR, NB, and RF) only accept the drug pairs with all features available as input.

Overall, 173 drug pairs were randomly divided into training set (approximately two-thirds, 115 drug pairs) and independent validation set-I (approximately one-third, 58 drug pairs) by keeping the original prevalence, which resulted in synergistic/antagonistic ratios of 85/30 and 42/16 in the training and validation sets, respectively (Supplementary Tables S3, S4).

To further verify the model performance of our developed model, we employed combination drugs used in TCGA project (The Cancer Genome Atlas Research Network et al., 2013). Specifically, we extracted the medical information of patients from The Cancer Genome Atlas (TCGA) project with the R package RTCGA^5^. Most of the patients were administered more than one drug, showing the necessity of multidrug therapy (Supplementary Figure S1). We consider that these patients had all undergone combinatorial therapy with synergistic effects. We screened out 659 patients who took just two kinds of drug with an overlap of at least 5 days, including 90 different drug combinations (Supplementary Tables S3, S4). The 90 drug combinations pairs were use as the independent validation-II.

(2) Feature selection: To compare the model performance with different combinations composed of seven preliminary features, XGBoost model were built with different feature combination, yielding 127 (i.e., $\sum_{i=1}^{7}C_{7}^{i}=127$) XGBoost models. The model performance of 127 XGBoost models were evaluated base on the average accuracy from 50 time of fivefold CV. The optimized feature combination was determined by the corresponding XGBoost model with highest accuracy, which was used as the final model for further analysis.

(3) Model evaluation: Six performance metrics were used including AUC, accuracy, sensitivity, specificity, negative predictive value (NPV), and positive predictive value (PPV) to evaluate the models. Synergistic combinations were classified as positive while antagonistic combinations were classified as negative. For training set, the average value of each performance metrics based on 50 runs of fivefold CV were presented. For independent validation set-I, six performance metrics were generated and further compared with the CV results, which was used to investigate whether the built model suffered over-fitness. To further investigate whether the XGBoost model performance was better than chance, a permutation test by using Y-scrambling strategy was implemented. Specifically, 2,000 permuted datasets were generated for the training set, in which the effect of drug pairs was randomly scrambled. For each permutation, the accuracy was calculated. Then, the *p-*value was calculated to assess the probability of the accuracy based on real data obtained by chance. For independent validation set-II, only the sensitivity was calculated since the comparison drug pairs are all synergistic.

(4) Comparison with state-of-the-art methods: To further compare the model performance of XGBoost with the state-of-the-art methods, four classifiers including RF, LR, NB classifier, and DeepSynergy (Preuer et al., 2018). The default parameters were used for LR, and NB with sklearn package in Python v3.5. For RF, we tested different numbers of estimators (trees) and features considered in each split. The performance is not well correlated with the hyperparameters. Thus, the performance of RF presented is generated based on default parameters. For DeepSynergy, 14 drug pairs are overlapped in the validation set-II and labeled with yellow background in Supplementary Table S7. DeepSynergy and our XGBoost were employed to compare their model performance with these drug pairs.

### Model Interpretation

#### Applicability Domain of the Developed XGBoost Model

Since the drug combination pairs curated cover a wide spectrum of different therapeutic categories, a defined applicability domain would be helpful for further application for various purpose. Therefore, those drug pairs with 50 correct or incorrect predictions were extracted based on the average accuracy of 50 runs of fivefold CV and further classified according to the second level of WHO Anatomic Therapeutic Class (ATC^6^) (Skrbo et al., 2004). Fisher’s exact tests were performed on these drug pairs for each drug category. The odds ratio is calculated by dividing the ratio of a certain kind of drug in drug pairs with correct prediction to all drugs with correct prediction on the one hand by the ratio of a certain kind of drug in drug pairs with incorrect prediction to all drugs with incorrect prediction on the other.

#### Pathway Analysis

To determine the association between predictive accuracy and biological relevance of the drug targets, the targets belonging to those drug pairs with 50 correct or incorrect predictions stated above were extracted and mapped to pathways in KEGG for enrichment analysis, respectively (Kanehisa et al., 2016). The enrich pathways were adjusted *p*-values less than 0.01 were considered as statistically significant pathways.

### Code Availability

The codes used for the generation of these features have been uploaded in https://github.com/514419407/Five-feature-Model-for-Predicting-the-Effects-of-Drug-Combinations-Built-by-XG Boost.git. XGBoost model was constructed by the xgboost package in Python. Other models built by other classifiers (LR, NB, and RF) were constructed by the sklearn package in Python. The xgboost and sklearn packages can be downloaded from https://pypi.org/. The values of all key hyperparameters of different algorithms are in Supplementary Table S5.

## Results

### Feature Selection

**FIGURE 2:**
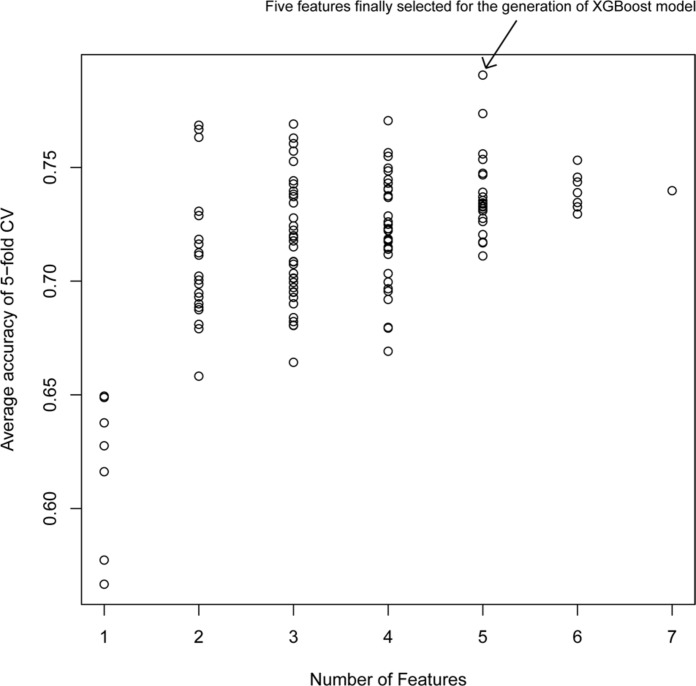
Model performance of XG models with different feature combinations: The average accuracy of 50 runs of cross-validation (CV) was calculated for different XG models.

Figure 2 shows the average accuracy from 50 repetitions of the fivefold CV for the feature selection process in the XGBoost models. A total of 127 ($i.e.,\sum_{i=1}^{7}C_{7}^{i}=127$) XGBoost models were developed based on the different combination of the seven features. The performance of all XGBoost models roughly tend to be stable after the size of features combination reached five; further increasing the number of features did not change the model performance or slightly decreased the performance. Thus, the five features with the highest accuracy were selected for the construction of the XGBoost model. The optimized five features included DID, ADRID, BPS, SMA, and separation score.

**FIGURE 3:**
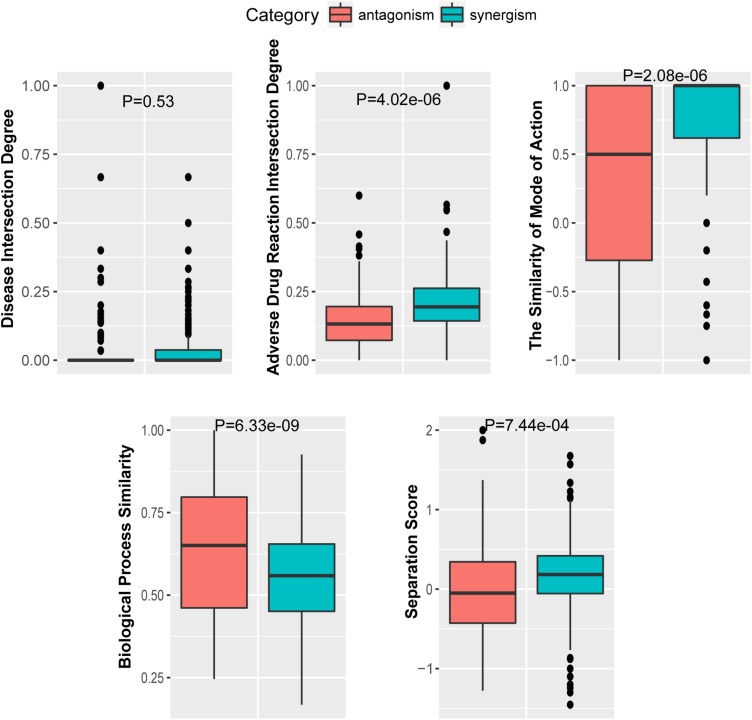
The *t*-test for the five optimized features.

**FIGURE 4:**
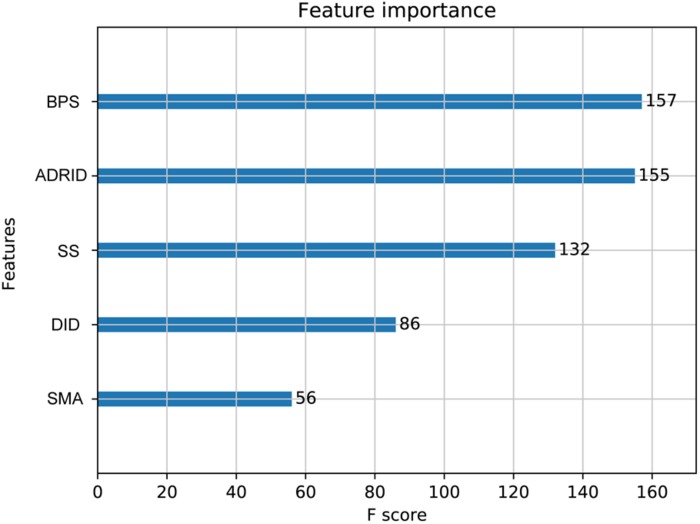
Feature importance contributed to the XGBoost model measured by *F*-score: The average *F*-score of each model is displayed from 50 repetitions of the fivefold cross-validation (CV) carried out in the training set. Features in order of their contributions from large to small are as follows: BPS, biological process similarity; ADRID, adverse drug reaction intersection degree; SS, separation score; DID, disease intersection degree; SMA, the similarity of mode of action.

To further investigate the performance contribution of each optimized features, the performance of the models constructed with different five feature combinations (one feature alone, leaving one feature out, and all five features) by the XGBoost classifier (Table 1). The results show that, among the metrics used for model evaluation, which include AUC, sensitivity, specificity, PPV, NPV, and accuracy, sensitivity achieved the best result in all models. The model with all the features showed the best performance, especially for specificity, which was much higher (at least 0.2) than those of the other models used in the comparison. Even for the SMA, the feature with the lowest *F*-score, the performance of all the leave-one-feature-out models was far behind that of the model built with all five features, showing the necessity of including all features in our model. The similar pattern was also observed based on Fisher’s exact test. All these features were found to differ significantly between synergistic drug pairs and antagonistic drug pairs (*t*-test, *p* < 0.05), except for in the DID (*t*-test, *p* = 0.53) in the training set (Figure 3 and Supplementary Table S6). Synergistic drug pairs show significantly higher ADRID, the SMA, and separation score, while showing significantly lower BPS (*t*-test, *p* < 0.05). The contribution of each feature to the XGBoost classifier is measured according to the intrinsic criterion of the XGBoost model, *F*-score (Chen and Guestrin, 2016) (Figure 4). The DID shows no significant difference between synergistic drug pairs and antagonistic drug pairs which is similar to its low contribution to the XGBoost classifier.

**TABLE 1:** Performance of models constructed with different feature combinations (one feature alone, leave one feature out, and all features) by the XGBoost classifier.

**Features**	**AUC**	**Sensitivity**	**Specificity**	**PPV**	**NPV**	**Accuracy**
DID	0.46	0.79	0.03	0.70	0.53	0.65
ADRID	0.57	0.82	0.08	0.72	0.50	0.64
BPS	0.66	0.89	0.37	0.74	0.51	0.62
SMA	0.55	0.86	0.38	0.73	0.40	0.65
SS	0.60	0.87	0.30	0.75	0.48	0.56
No DID	0.74	0.89	0.46	0.73	0.62	0.70
No ADRID	0.71	0.90	0.30	0.73	0.55	0.69
No BPS	0.70	0.90	0.24	0.75	0.56	0.67
No SMA	0.73	0.92	0.40	0.74	0.58	0.68
No SS	0.73	0.91	0.43	0.73	0.59	0.68
All	0.77	0.95	0.63	0.82	0.67	0.79

*DID, disease intersection degree; ADRID, adverse drug reaction intersection degree; BPS, biological process similarity; SMA, similarity of mode of action; SS, separation score. PPV, positive predictive value: TP/(TP+FP). NPV, negative predictive value: TN/(TN+FN). The average metrics of each model are displayed from 50 repetitions of the fivefold cross-validation (CV) carried out in the training set. The column names are the models made up of different combinations. The first five rows are models constructed with one feature alone; the middle five rows are models constructed when leaving one feature out; the last row is the model constructed with all five features.*

### Model Performance for Validation Set-I

**FIGURE 5:**
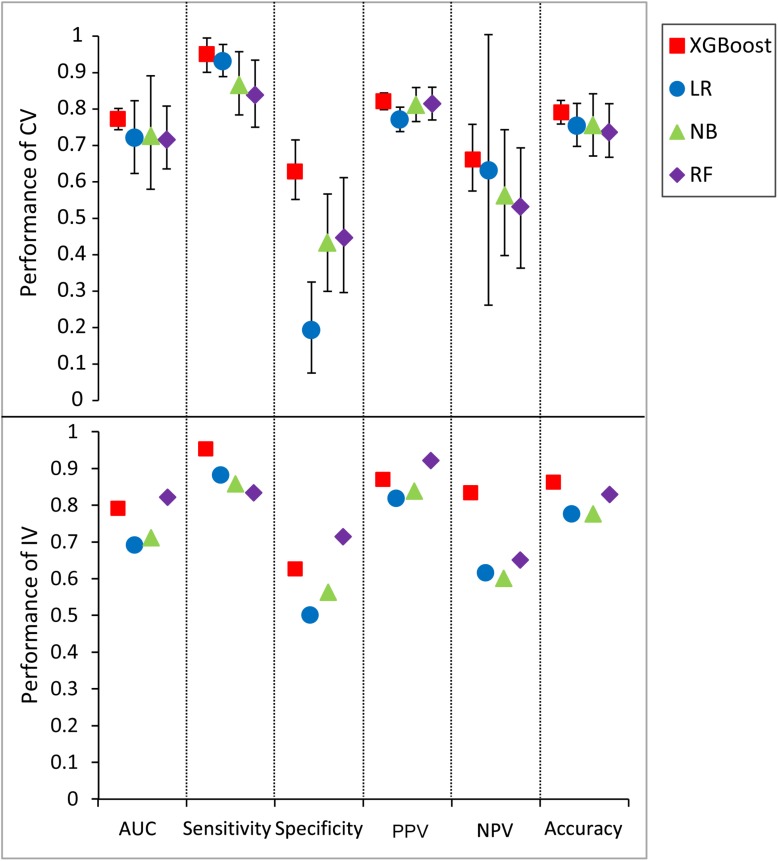
Predictive values and standard deviation for six different metrics [AUC, sensitivity, specificity, positive predictive value (PPV), negative predictive value (NPV), accuracy] for the models constructed by different classifiers for both cross-validation (CV) and independent validation (IV). LR, logistic regression; NB, naïve Bayesian; RF, random forest.

An extensive comparison of models built by XGBoost and other models was performed with all five features (see section “Materials and Methods”). Figure 5 shows the six performance metrics based on 50 runs of in fivefold CV and independent validation (IV) for models built with different classifiers (Supplementary Tables S6, S7). The standard deviations of all CV metrics in the model built by XGBoost are all lower than those built by other classifiers when the values of all CV metrics in the XGBoost model are greater than those in models built by other classifiers including RF, LR, and NB. A similar trend can also be observed for the other four IV performance metrics. For example, the values of four IV metrics in the XGBoost model are greater than those in models built by other classifiers. The values of accuracy in the XGBoost model in both CV and IV are at least 0.03 higher than those in models built by other classifiers. The performance ranks of the models on the IV set in terms of sensitivity and PPV are exactly consistent with the CV results. Since F1 score [2^*^((precision^*^recall)/(precision+recall))] conveys the balance between the precision and the recall, we also compared the values of F1 score among different models. The values of F1 score in the XGBoost model in both CV and IV are at least 0.025 higher than those in models built by other classifiers (Supplementary Table S8), with more true positives and fewer false negatives.

**FIGURE 6:**
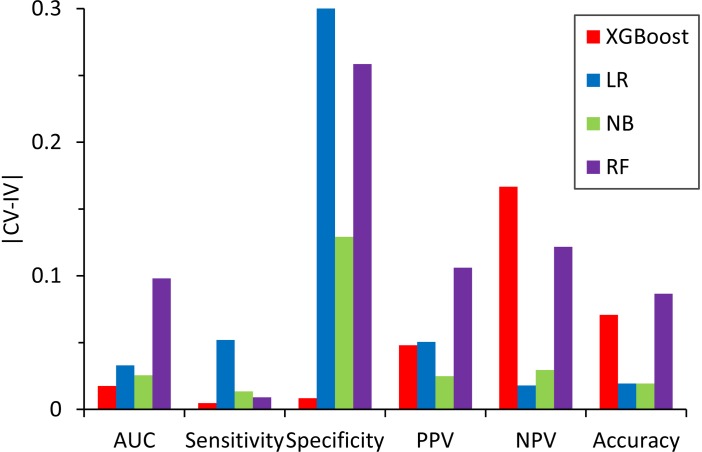
Absolute difference, |CV – IV|, between the predictive performance for the six performance metrics in the fivefold cross-validation of the training set and the independent validation set for the models constructed by different classifiers.

We also compared the difference in the six-performance metrics between the CV and IV (Figure 6), denoted as |CV − IV|, for the models constructed using four classifiers. The |CV − IV| value measures the concordance; that is, a large |CV − IV| value indicates either overtraining in the training model (CV > IV) or an unreliable extrapolation (IV > CV), since the performance of the internal validation should not be significantly better than that of the external validation. In addition to the best overall performance in both CV and IV, the XGBoost model also has the smallest |CV − IV| values of the metrics (AUC, sensitivity, and specificity) among the different models.

**FIGURE 7:**
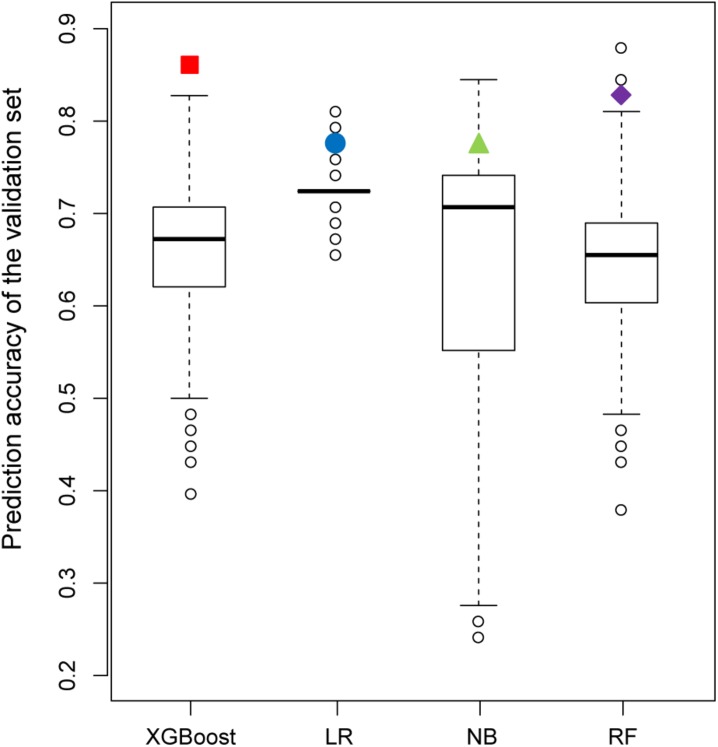
Comparison of the prediction accuracy of the validation set between 2,000 models derived from a permutation test (randomly permuted) and the real dataset (colored solid dots).

Figure 7 shows the results of the permutation tests to assess whether the models predict the validation set better than would be expected by chance alone (see section “Materials and Methods”). If the predictive performance of a model measured by the real training set is not greater than that measured by the permutated training sets, we can conclude that the model measured by the real training set performs no better than the random results. Similar to the findings described in the previous section, the XGBoost model achieved the best performance in permutation tests. Unlike XGBoost, some of the values of prediction accuracy of the validation set derived from permutation tests were higher than those of the validation set derived from the real dataset in all other models.

### Model Evaluation by Validation Set-II

To further confirm the performance of XGBoost, we tested the validation set obtained from TCGA with the XGBoost model. Of the 90 drug pairs involved in patients who underwent combinatorial therapy with a synergistic effect in TCGA (see section “Materials and Methods”), 61 drug pairs contained at least one feature in the XGBoost model. The XGBoost model classified these drug pairs with accuracy of 0.787 (Supplementary Table S7). These 61 drug pairs were used in 610 patients with 27 cancer types, with accuracy of over 0.94 calculated by the number of patients in TCGA, further demonstrating the robustness of our model.

To further validate the classification ability of the five-feature XGBoost model, we compared the prediction ability of the prediction ability between the five-feature XGBoost model and DeepSynergy. The original data profiles of the five-feature XGBoost model and DeepSynergy are different. To compare the prediction performance between the five-feature XGBoost model and DeepSynergy, we detected 14 overlapped drug pairs between the validation set-II of the five-feature XGBoost model and the prediction dataset of DeepSynergy since TCGA data are focused on cancer therapy. We displayed the predicted accuracy of the 14 overlapped drug pairs in 38 cell lines in DeepSynergy and in validation set-II. The highest accuracy could reach to 0.86 by using DeepSynergy, which is comparable to the accuracy (0.787) generated by XGBoost (Supplementary Table S9).

### Distribution of Predicted Effectiveness by the Developed XGBoost Model

**FIGURE 8:**
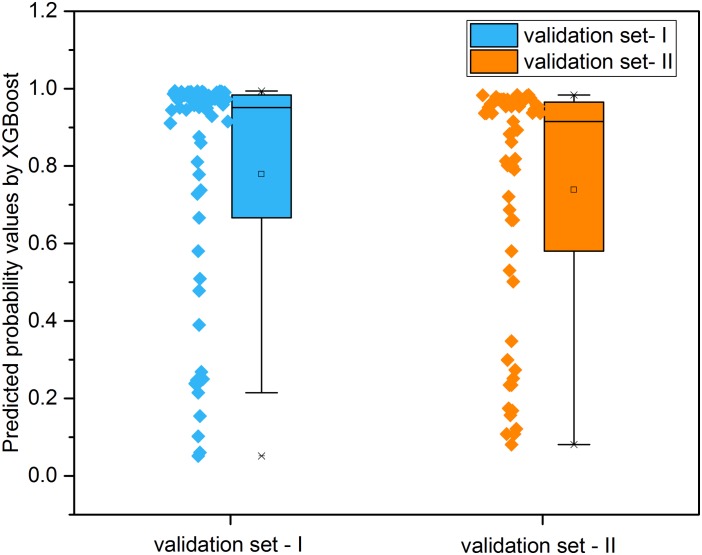
The distribution of predicted probability values derived from XGBoost models for the two independent validation sets.

Figure 8 illustrated the distribution of possibility values for the two independent validation sets (Supplementary Tables S6, S7). The average possibility value of validation set-I and validation set-II since the drug pairs are 0.7788 ± 0.3074 and 0.7384 ± 0.3079. The large standard deviation indicated that the possibility values could be utilized to quantitatively reflect the effectiveness of drug combination pairs. Specifically, the scale of possibility is in a range of 0 to 1. The bigger possibility values indicated the higher synergistic effect. The lower possibility values mean the stronger antagonistic effect of drug pairs. The drug pairs with addictive effect were with possibility values around 0.5.

### Applicability Domain of XGBoost Models

We then aimed to determine whether our model is able to classify drug pairs varied in different drug categories (see section “Materials and Methods”). Of the 822 drug pairs that we collected, the effectiveness of 745 drug pairs was correctly predicted at least once, while the effectiveness of 218 drug pairs was wrongly predicted at least once. The effectiveness of 604 drug pairs was correctly predicted in all 50 iterations, while the effectiveness of 77 drug pairs was wrongly predicted in all 50 iterations, showing the stability of the five-feature XGBoost model.

Drugs belonging to drug pairs with consistent prediction in all 50 iterations (both correct and incorrect predictions) were extracted to measure the predictive accuracy for different therapeutic categories. Among the 14-main anatomical/ pharmacological groups classified based on WHO Anatomic Therapeutic Class (ATC, see text footnote 6), for drugs belonging to five groups, there are significant increases (odds ratio < 1) or reductions (odds ratio > 1) on their predictive accuracy (Fisher’s exact test, *p* < 0.05) (Table 2, see section “Materials and Methods”). Specifically, among the drugs belonging to five groups, for antineoplastic and immunomodulating agents (abbreviated to L) and anti-infectives for systemic use (abbreviated to J), there is a significantly higher proportion of drugs in drug pairs with correctly predicted effectiveness than that of drugs in drug pairs with incorrectly predicted effectiveness (Fisher’s exact test, *p* < 0.01; odds ratio < 1); for the drugs belonging to other three groups, there is a significantly lower proportion of drugs in drug pairs with correctly predicted effectiveness than that of drugs in drug pairs with incorrectly predicted effectiveness (Fisher’s exact test, *p* < 0.01; odds ratio > 1).

**TABLE 2:** Association of prediction accuracy and drug classification according to ATC codes by the stratified fivefold cross-validation.

**Anatomical main group**	**Abbreviation**	**Odds ratio**	***P*-value**	**#Drugs**
Antineoplastic and immunomodulating agents	L	0.20	0.00	218
Nervous system	N	2.19	0.00	151
Various	V	4.43	0.00	27
Anti-infectives for systemic use	J	0.41	0.01	86
Alimentary tract and metabolism	A	1.84	0.03	50
Musculo-skeletal system	M	2.00	0.07	24
Respiratory system	R	1.77	0.10	32
Genito urinary system and sex hormones	G	1.61	0.19	33
Blood and blood forming organs	B	0.27	0.24	28
Antiparasitic products, insecticides and repellents	P	1.68	0.27	21
Dermatologicals	D	1.16	0.60	48
Sensory organs	S	1.09	0.76	60
Cardiovascular system	C	1.04	0.89	99
Systemic hormonal preparations, excl. sex hormones and insulins	H	0.86	1.00	8

*The table is sorted according to *P*-values from low to high. The employed drugs belong to drug pairs with consistent prediction in all 50 iterations (both correct and incorrect predictions).*

### Associating Pathways With the Potential of the Five-Feature XGBoost Model

We next investigated whether our model can classify synergistic vs. antagonistic drug pairs with targets belonging to different pathways (see section “Materials and Methods”). We enriched the targets of drugs in correctly and incorrectly predicted drug pairs to 139 and 96 KEGG pathways (Bonferroni, *p-*value < 0.01), respectively (Kanehisa et al., 2016). Forty-three pathways exclusively belonged to the correctly predicted drug pairs (Table 3). The results of pathway analysis correspond to the results of drug category analysis. A number of pathways are associated with antineoplastic and immunomodulating agents, anti-infectives for systemic use including for malaria (Nosten and White, 2007), and bacterial invasion of epithelial cells.

**TABLE 3:** Forty-three pathways exclusively belonging to correctly predicted drug pairs.

**Pathway name**	**#Gene**	***p*-Value**
Proteasome	40	3.06E-54
Cytokine–cytokine receptor interaction	59	3.10E-31
Jak-STAT signaling pathway	35	2.27E-18
Epithelial cell signaling in *Helicobacter pylori* infection	24	2.72E-17
Leukocyte transendothelial migration	29	2.25E-16
NOD-like receptor signaling pathway	21	2.76E-15
Arrhythmogenic right ventricular cardiomyopathy (ARVC)	22	5.84E-14
Shigellosis	20	1.53E-13
Hematopoietic cell lineage	21	3.44E-11
African trypanosomiasis	14	1.37E-10
Malaria	16	2.57E-10
Rheumatoid arthritis	20	6.71E-10
Adherens junction	18	9.96E-10
Base excision repair	13	1.15E-09
PPAR signaling pathway	16	5.14E-08
Dorso-ventral axis formation	10	1.70E-07
Bacterial invasion of epithelial cells	15	4.84E-07
RIG-I-like receptor signaling pathway	15	5.97E-07
Wnt signaling pathway	21	1.29E-06
Protein digestion and absorption	15	3.99E-06
Arginine and proline metabolism	12	1.23E-05
Axon guidance	18	1.60E-05
Parkinson’s disease	17	9.34E-05
Caffeine metabolism	5	9.90E-05
One carbon pool by folate	7	0.0001
Nucleotide excision repair	10	0.0001
Taste transduction	10	0.0006
Vibrio cholerae infection	10	0.0009
Tyrosine metabolism	8	0.0054
Type I diabetes mellitus	8	0.0078
Protein processing in endoplasmic reticulum	16	0.0094
ECM–receptor interaction	11	0.0105
Terpenoid backbone biosynthesis	5	0.0115
Nicotinate and nicotinamide metabolism	6	0.0127
Vitamin digestion and absorption	6	0.0127
Fat digestion and absorption	8	0.0129
DNA replication	7	0.0176
Allograft rejection	7	0.0189
Renin–angiotensin system	5	0.0189
Graft-versus-host disease	7	0.0378
Autoimmune thyroid disease	8	0.0378
Pyruvate metabolism	7	0.0378
Glycerophospholipid metabolism	10	0.0378

## Discussion

The five-feature XGBoost model is an important advance for the classification of synergistic and antagonistic drug pairs. Classifying synergistic vs. antagonistic drug pairs experimentally is time-consuming and labor-intensive. *In silico* methods can thus be of tremendous benefit in this field of study. In this paper, we propose a model for efficiently classifying synergistic and antagonistic drug pairs. Its comparison with other models showed that it confers major advantages in accurately classifying synergistic vs. antagonistic drug pairs in combination, both with and without the existence of all five features.

With the extremely low |CV − IV| value of sensitivity and the highest values in sensitivity and accuracy received from the XGBoost classifier, the five-feature XGBoost model shows much greater ability to predict the effects of combinatorial therapies with synergistic effects than those with antagonistic effects. Thus, our model is reliable for use as a filter to generate candidates of synergistic drug pairs. For example, the combination of caffeine and hexobarbital is an antagonistic drug pair that was wrongly classified as a synergistic drug pair by our model. This may have been due to the lack of feature values (DID and ADRID) in this drug pair.

According to our research, our model is preferable to classify synergistic vs. antagonistic drug pairs composed of antineoplastic and immunomodulating agents, anti-infectives for systemic use (Table 2). This may be due to the fact that cancer patients receive combinatorial drug therapy with targeted drugs in some circumstances (Al-Lazikani et al., 2012). The results of pathway analysis correspond to the results of drug category analysis. For example, malaria is treated by anti-infectives for systemic use and a pathway in KEGG belonging to the correctly predicted drug pairs. The reason for the excellent performance of the five-feature XGBoost model in malaria is according to the performance in anti-infectives for systemic use (Table 2) and malaria pathway (Table 3) that our prediction model follows the rules of combinatorial therapy for malaria of reducing the risk of treatment failure and reducing the side effects (Nosten and White, 2007).

Besides the advantages stated above, XGBoost can be constructed and performs prediction when drug pairs do not contain all five features, so it is more practical than other models as, among our 822 collected known drug pairs, only 173 contain all five features (Supplementary Table S2).

The five-feature XGBoost model contains relatively few features compared with other models (Sun et al., 2015). However, the features in our model are ubiquitous among drugs and other molecules potentially available for medical usage with vital medical significance. Intriguingly, our synergistic drug pairs show no significant difference from antagonistic drug pairs according to DID. This may be because not all the indications of the drug have been detected yet. In addition, although the SMA uses more precise information (promotive/inhibitory drug–target and protein–protein relationships) than other features, it makes the smallest contribution to our model. This may be due to the fewer related data.

It is worthwhile to consider some additional studies to further our knowledge and improve the prediction results from this study. First, the current *in silico* drug combination models are mainly focused on the field of oncology. There is thus a lack of *in silico* models to explore the opportunities for using drug combinations in other therapeutic categories such as pediatric and infectious diseases. Second, numerous accumulative biological datasets have been generated and become widely available, so a comprehensive assessment of the predictive power of diverse biological profiles is imperative to provide useful information for further model development. Third, the fine-tuning hyperparameters of machine-learning algorithm such as RF may provide improved model performance, however, it is not the focus of current study. Final, some novel algorithms for drug combination effectiveness prediction such as TreeCombo is worth exploring for better prediction results (Janizek et al., 2018).

## Conclusion

In conclusion, we applied one machine-learning methodology, XGBoost, to classify the effects of drug combinations, which was greatly successful. In future work, deep learning algorithm such as RNN is also worth investigating for potential performance improvement. Although some other important features such as gene expression are not incorporated into our model (Sun et al., 2015), it may make a major contribution to predicting the effects of drug combinations.

## Author Contributions

ZL and TS designed the study. ZL and XJ performed the data analysis and wrote the manuscript. TS, XJ, ZL, and WT revised the manuscript. All authors read and approved the final manuscript.

## Disclaimer

The views presented in this article do not necessarily reflect current or future opinion or policy of the United States Food and Drug Administration. Any mention of commercial products is for clarification and not intended as an endorsement.

## Conflict of Interest Statement

The authors declare that the research was conducted in the absence of any commercial or financial relationships that could be construed as a potential conflict of interest.

## Footnotes

1 http://www.cls.zju.edu.cn/dcdb/index.jsf (accessed April, 2019).

2 http://bidd.nus.edu.sg/group/cjttd/TTD_HOME.asp

3 https://www.ncbi.nlm.nih.gov/pubmed/

4 http://opennlp.apache.org/

5 https://rtcga.github.io/RTCGA/

6 http://www.whocc.no/atcddd/

## Supplementary Material

FIGURE S1 The distribution of patient sample size with different numbers of drugs during medical therapy from TCGA.

TABLE S1 Information on 167 drug combinations retrieved from PubMed.

TABLE S2 Features and real effectiveness of 822 known drug pairs.

TABLE S3 Patients who took just two kinds of drugs with an overlap of at least 5 days from TCGA.

TABLE S4 Tumor types included in Supplementary Table S2.

TABLE S5 Key hyperparameters used in different models.

TABLE S6 Features and real effectiveness used in the training set and validation set-1.

TABLE S7 Features, real effect, and predicted effect of 61 drug pairs from TCGA based on the five-feature XGBoost model.

TABLE S8 The values of F1 score in CV and IV.

TABLE S9 Accuracy of the 14 overlapped drug pairs in 38 cell lines in DeepSynergy and in validation set-2.
