# Supplementary figures and images for: Five-Feature Model for Developing the Classifier for Synergistic vs. Antagonistic Drug Combinations Built by XGBoost
Source: Front Genet. 2019 Jul 9;10:600. doi: 10.3389/fgene.2019.00600 (PMC6629777; doi:10.3389/fgene.2019.00600)

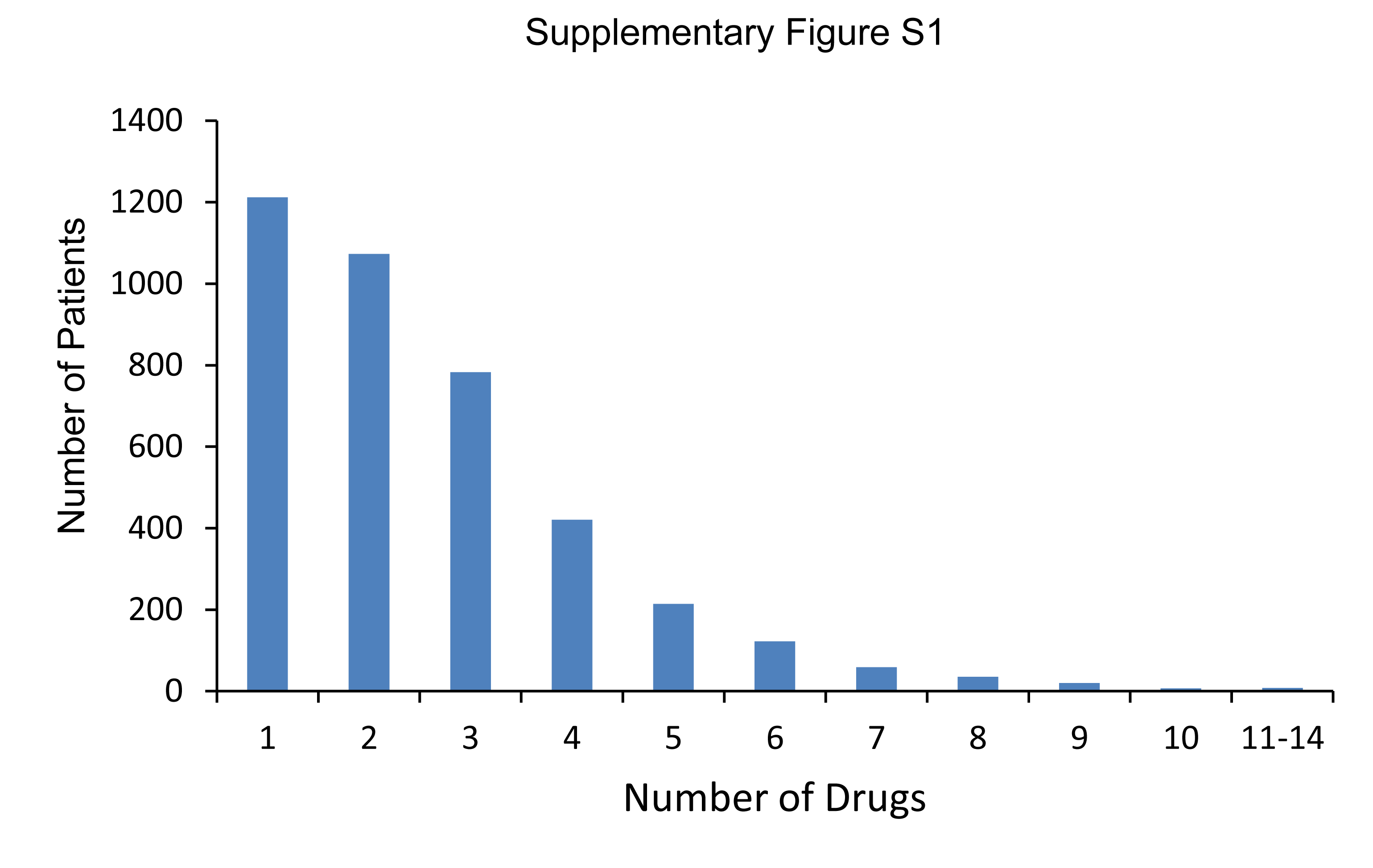

Supplement: FIGURE S1 — The distribution of patient sample size with different numbers of drugs during medical therapy from TCGA. [file Image_1.tif]
